# The contribution of TRPC1, TRPC3, TRPC5 and TRPC6 to touch and hearing

**DOI:** 10.1016/j.neulet.2015.10.052

**Published:** 2016-01-01

**Authors:** Jane E. Sexton, Terri Desmonds, Kathryn Quick, Ruth Taylor, Joel Abramowitz, Andy Forge, Corné J. Kros, Lutz Birnbaumer, John N. Wood

**Affiliations:** a Molecular Nociception Group, Wolfson Institute for Biomedical Research, University College London, London WC1E 6BT, UK; b Sussex Neuroscience, School of Life Sciences, University of Sussex, Brighton BN1 9QG, UK; c UCL Ear Institute, 332 Gray's Inn Road, London WC1X 8EE, UK; d Neurobiology Laboratory, National Institute of Environmental Health Sciences, 111 TW Alexander Dr., Research Triangle Park, N.C. 27709, USA; e Dept. Otorhinolaryngology, University Medical Center Groningen, University of Groningen, 9700 RB Groningen, The Netherlands

**Keywords:** DKO, double knockout mice, QuadKO, quadruple knockout mice, Mechanosensation, TRP channels, Touch

## Abstract

Transient receptor potential channels have diverse roles in mechanosensation. Evidence is accumulating that members of the canonical subfamily of TRP channels (TRPC) are involved in touch and hearing. Characteristic features of TRP channels include their high structural homology and their propensity to form heteromeric complexes which suggests potential functional redundancy. We previously showed that TRPC3 and TRPC6 double knockout animals have deficits in light touch and hearing whilst single knockouts were apparently normal. We have extended these studies to analyse deficits in global quadruple TRPC1, 3, 5 and 6 null mutant mice. We examined both touch and hearing in behavioural and electrophysiological assays, and provide evidence that the quadruple knockout mice have larger deficits than the TRPC3 TRPC6 double knockouts. Mechano-electrical transducer currents of cochlear outer hair cells were however normal. This suggests that TRPC1, TRPC3, TRPC5 and TRPC6 channels contribute to cutaneous and auditory mechanosensation in a combinatorial manner, but have no direct role in cochlear mechanotransduction.

## Highlights

• The contribution of multiple TRPC channels on mechanosensory function is demonstrated.
• We observe a critical role for TRPC channels in touch sensation.
• TRPC channels contribute to cutaneous and auditory mechanosensation in a combinatorial manner, but have no direct role in cochlear mechanotransduction.

## 1 Introduction

The mechanisms underlying mechanotransduction in mammals are incompletely understood. Piezo2 has been shown to be essential for light touch sensitivity, in mechanical allodynia in neuropathic conditions and produces a mechanically activated, rapidly adapting current [5], [8], [24], [33]. Transient receptor potential (TRP) channels are a superfamily of structurally homologous cation channels which have diverse roles in sensory functions. We have previously discussed the extensive evidence implicating TRP channels in mechanosensory roles in many different species, including TRPA1 which has an important role in cutaneous mammalian mechanosensation [2], [18], [21], [32].

We also reported previously, a combinatorial role for TRPC3 and TRPC6 in mediating normal touch and hearing [23]. The canonical subfamily of TRP (TRPC) channels have known roles in mechanosensory function in mammalian systems including the cardiovascular system [7] and the kidneys [16] and there is an increasing pool of evidence implicating members of the TRPC subfamily in cutaneous mechanosensory functions. In the DRG, TRPC1, TRPC3 and TRPC6 are the most abundantly expressed TRPC subunits and their expression has been observed in most sensory neurons in adult mice [10], [23]. In addition, TRPC5 has been found to be localised to small and medium diameter sensory neurons [34]. A single cell RNA sequencing study also determined a non-peptidergic subset of neurons which express all four TRPC subunits [30] meaning there is substantial potential for interaction between different combinations of these TRPC subunits. TRPC1 and TRPC6 are coexpressed with TRPV4 in dorsal root ganglia (DRG) and it has been proposed that they may act in concert to mediate mechanical hypersensitivity in neuropathic and inflammatory pain states [1]. TRPC1 null animals show a decrease in sensitivity to innocuous mechanical stimuli and show a reduction in down hair Aδ and slowly adapting Aβ fibre firing in response to innocuous mechanical stimulation [11]. TRPC1 and TRPC5 confer sensitivity to osmotically induced membrane stretch in cultured DRG neurons and HEK293 cells, respectively [13], [28]. TRPC6 is also activated by membrane stretch while both TRPC5 and TRPC6 activity is blocked by a tarantula toxin known to inhibit mechanosensitive channels [27]. In addition, TRPC channels are ubiquitously expressed in the inner ear in structures including the organ of Corti and the spiral and vestibular ganglia [29] suggesting that, in addition to TRPC3 and TRPC6, there is potential for other TRPC subunits to play a mechanosensory role in hearing.

In the current study we extended our analysis of TRPC channels and their role in mechanosensation. TRP channels are known to function in heteromeric complexes and are believed to show functional redundancy. In order to minimise the effects of compensation mechanisms which these qualities confer, we progressed from investigating sensory function in TRPC3 and TRPC6 double knockout animals (both knockout, or DKO, animals) to looking at animals with global knockouts of TRPC1, TRPC3, TRPC5 and TRPC6 channels (quadruple knockout, or QuadKO, animals). We previously provided evidence that TRPC3 channels contribute to mechanotransduction in some cell lines, but not others, consistent with some role for TRPC channels in mechanotransduction [23]. Here we provide further evidence of a combinatorial role for TRP channels in mechanosensation.

## 2 Results

### 2.1 TRPC1, TRPC3, TRPC5 and TRPC6 knockout animals have selective deficits to light touch stimuli but normal responses to thermal stimuli

**Fig. 1:**
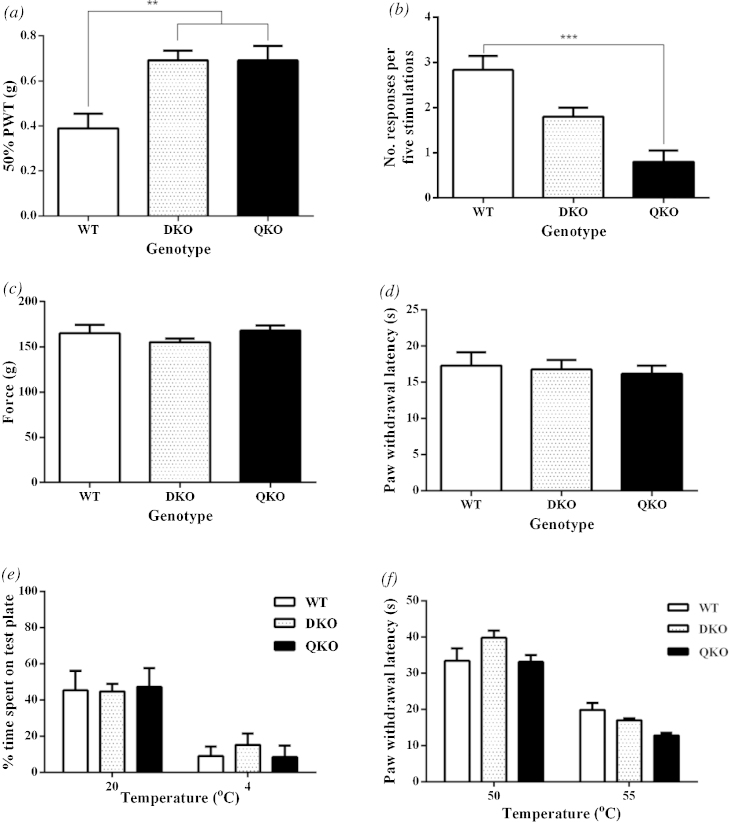
Modality specific sensory deficits in multiple KO animals. (a) DKO (0.69 g ± 0.04 g) (*n *= 10) and QuadKO (0.69 g ± 0.06 g) (*n *= 10) show an increase in 50% withdrawal threshold compared to WT (0.39 g ± 0.06 g) (*n *= 10) but no difference is seen between the two test groups. (b) QuadKO (0.8 ± 0.25) (*n *= 10) show a stepwise decrease in the percentage responses to a dynamic cotton swab stimulus compared to WT (2.83 ± 0.31) (*n *= 6) and DKO (1.8 ± 0.2) (*n *= 10). (c) No difference was observed in sensitivity to noxious mechanical force between groups (WT *n *= 6, DKO *n *= 10, QuadKO *n *= 10). (d) No difference was observed in sensitivity to noxious heat between groups (*n *= 6 all groups). (e) No difference was observed in sensitivity to a hot plate between groups (*n *= 6 all groups). (f) No difference was observed between groups of the time spent on a noxious cold plate (4 °C) following an acclimatisation session (20 °C) (*n *= 6 all groups). Data are shown as Mean ± SEM with one-way ANOVA with Tukey post test (a,c,d); Kruskal Wallis Test with Dunn’s post test (b) and two-way ANOVA with Tukey post test (e,f) *p < 0.05; **p < 0.01; ***p < 0.001; ****p < 0.0001.

We found that QuadKO animals showed deficits in light touch sensitivity compared to WT animals, shown by an increase from 0.39 g to 0.69 g in the 50% withdrawal threshold to von Frey hairs (WT v. DKO *p *= 0.003; WT v. Quad KO *p *= 0.003; DKO v Quad KO *p *= 0.99; Fig. 1a) and a 41% decrease in the percentage response to a dynamic cotton swab application to the paw (WT v. DKO *p *= 0.20; WT v. Quad KO *p *= 0.0006; DKO v Quad KO *p *= 0.07; Fig. 1b). Interestingly, QuadKO animals did not show any difference in 50% withdrawal threshold compared to DKO animals but showed a decrease in the response to cotton swab stimulation compared to DKO, though this was not significant (*p *= 0.07).

Responses to high force mechanical stimuli, on the tail, were unimpaired in all groups (WT v. DKO *p *= 0.54; WT v. Quad KO *p *= 0.95; DKO v Quad KO *p *= 0.27; Fig. 1c).

Unimpaired responses to noxious heat stimuli in knockout animals (WT v. DKO *p *= 0.97; WT v. Quad KO *p *= 0.85; DKO v Quad KO *p *= 0.95; Fig. 1d, e) suggest that these TRPC channels are unlikely to be involved in transduction of noxious heat. We used a place preference paradigm to study QuadKO sensitivity to noxious cold temperatures. A baseline recording showed all groups spent ∼50% of the test session on the test plate; when the temperature was lowered to 4 °C, this dropped to ∼5% of the test session indicating all groups were aversive to the noxious cold temperature (WT v. DKO *p *= 0.83; WT v. Quad KO *p *= 0.99; DKO v Quad KO *p *= 0.81; Fig. 1f).

### 2.2 TRPC1, TRPC3, TRPC5 and TRPC6 knockout animals have impaired auditory and vestibular function

**Fig. 2:**
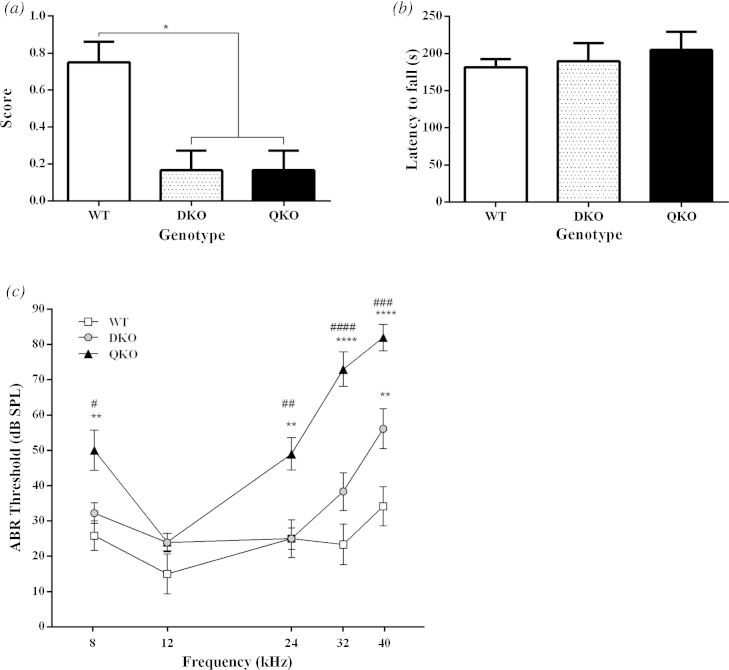
Impaired vestibular function seen in (a) trunk curl response (*n *= 6 all groups) but not in (b) rotarod test (*n *= 6 all groups) (c) Higher threshold responses to auditory pip-tone stimuli in Auditory Brainstem Response recordings in QuadKO (*n *= 5) compared to DKO (*n *= 9) and WT (*n *= 5) groups. Data are shown as Mean ± SEM with Kruskal–Wallis test with Dunn’s post test (a), one-way ANOVA with Tukey post test (b) and two-way ANOVA with Tukey post test (c); *p < 0.05; **p < 0.01; ***p < 0.001; ****p < 0.0001. (*denotes significance compared to WT; # denotes significance compared to DKO).

Using the trunk curl test [14], we found that QuadKO animals show some vestibular deficits which are comparable to deficits in DKO animals (WT v. DKO *p *= 0.03; WT v. Quad KO *p *= 0.03; DKO v Quad KO *p *= 0.99; Fig. 2a) but that TRPC multiple KO animals show latencies to fall from an accelerating rotarod that are comparable to those observed for WT and DKO mice (WT v. DKO *p *= 0.96; WT v. Quad KO *p *= 0.72; DKO v Quad KO *p *= 0.87; Fig. 2b) suggesting unimpaired motor coordination. As we reported previously, the role for the rotarod test in assessing vestibular function has been disputed as other studies have found that it does not always correlate with vestibular deficits presented by other relevant tests [19], [23]. Also, the trunk curl test is a rudimentary measure of vestibular function therefore more in depth tests would likely provide more information about the nature of these deficits [14].

Auditory brainstem response recordings (ABRs) were used to assess the auditory function of these animals where auditory pip tone stimuli are used to determine the threshold in decibels which is required to elicit a response at different frequencies. We found at frequencies of 8, 24, 32 and 40 kHz that QuadKO animals had a significantly higher response threshold than both WT and DKO animals (8 kHz, WT v. DKO *p *= 0.57; WT v. Quad KO *p *= 0.004; DKO v Quad KO *p *= 0.03; 12 kHz, WT v. DKO *p *= 0.34; WT v. Quad KO *p *= 0.43; DKO v Quad KO *p *= 0.99; 24 kHz, WT v. DKO *p *= 0.99; WT v. Quad KO *p *= 0.004; DKO v Quad KO *p *= 0.002; 32 kHz, WT v. DKO *p *= 0.05; WT v. Quad KO *p *= 0.0001; DKO v Quad KO *p *= 0.0001; 40 kHz, WT v. DKO *p *= 0.002; WT v. Quad KO *p *= 0.0001; DKO v Quad KO *p *= 0.0006; Fig. 2c).

### 2.3 TRPC1, TRPC3, TRPC5 and TRPC6 knockout animals have unimpaired mechano-electrical transducer currents in the hair cells of the inner ear

**Fig. 3:**
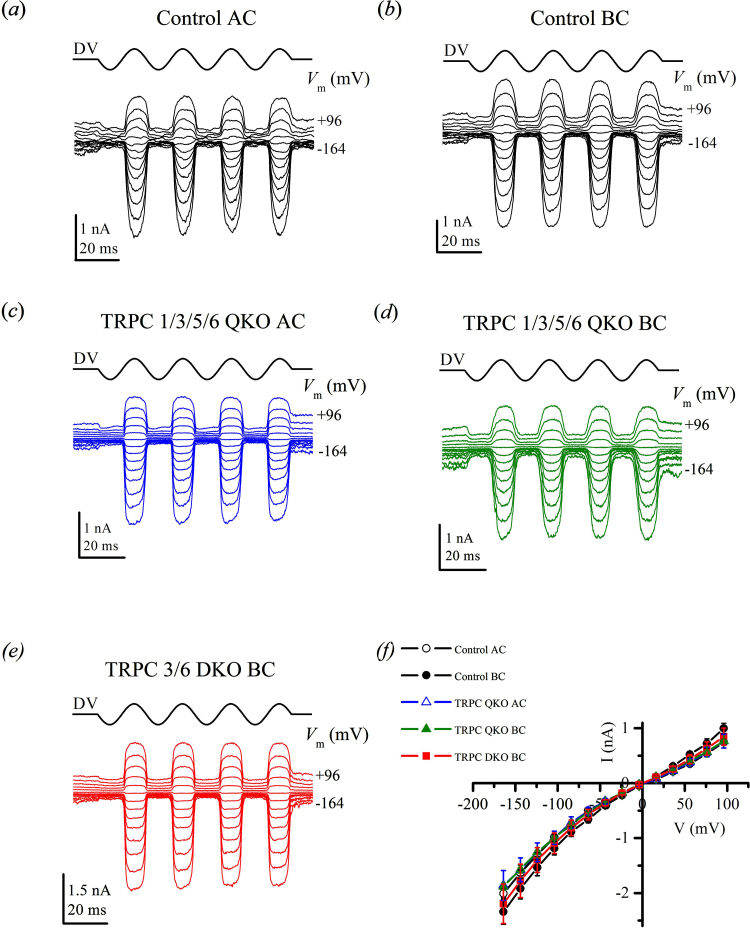
Unimpaired mechanotransduction in OHCs of QuadKO and DKO animals. (a–d) MET currents in response to 45 Hz sinusoidal force stimuli from a fluid jet. Holding potential was −84 mV and the membrane potential was stepped between −164 mV and +96 mV in 20 mV increments. Driver voltage (DV) amplitude was 40 V. Positive DV moves the hair bundles in the excitatory direction towards the kinocilium. (a) WT control OHC, postnatal day 2 + 1 (P2 + 1), mid-apical coil. (b) WT control OHC, P2 + 2 mid-basal coil. (c) QuadKO OHC, P2 + 1, mid-apical coil. (d) QuadKO OHC, P2 + 2, mid-basal coil. (e) DKO OHC, P2 + 1, mid-basal coil. (f) Current-voltage curves averaged from 5 mid-apical WT OHCs (black open circles), 4 mid-basal WT OHCs (black closed circles), 3 mid-apical QuadKO OHCs (blue open triangles); 2 mid-basal QuadKO OHCs (green closed triangles) and 3 mid-basal DKO OHCs (red squares). (For interpretation of the references to colour in this figure legend, the reader is referred to the web version of this article.)

Mechano-electrical transducer (MET) currents evoked by sinusoidal force stimuli in both the basal (associated with responses to high frequency stimuli) and apical (associated with responses to low frequency stimuli) coil of the cochlea, show normal amplitudes in outer hair cells (OHCs) of QuadKO animals, of comparable size to currents recorded from OHCs of matching WT control mice [12], [17] (Fig. 3a–d). MET currents of the QuadKO OHCs were similar in all respects to those of the WT control OHCs: currents reversed near zero mV, a fraction of the MET channels were open at rest and this fraction increased for depolarized membrane potentials due to a reduction in Ca^2+^-dependent adaptation [4].These observations suggest that the process of mechanotransduction in the cochlea is unaltered in knockout animals. Earlier data [23], suggested that MET currents in basal-coil OHCs of TRPC3/TRPC6 DKO OHCs were on average substantially smaller than those of WT controls. Further experiments using the same methods for MET current recording showed that it is possible to record large MET currents from TRPC3/TRPC6 DKO OHCs in the basal coil (Fig. 3e). The current–voltage curves (Fig. 3f) were similar between the five groups of OHCs being compared. For example, MET current size of OHCs at −104 mV (mean ± SEM) was: WT control apical coil: −983 ± 47 pA, *n *= 5; WT control basal coil: −1185 ± 121 pA, *n* = 4; QuadKO apical coil: −993 ± 120 pA, *n* = 3; QuadKO basal coil: −997 ± 37 pA, *n* = 2; DKO basal coil: −1091 ± 187 pA, *n* = 3. There were no significant differences (*p* > 0.05; ANOVA with Tukey post-test) between any of these groups. This negates our earlier finding of on-average smaller currents in basal-coil DKO OHCs. The previously observed diminished inward currents in basal-coil DKO OHCs may be explained by sub-optimal organotypic cultures. The present results do not support a role for TRPC channels in primary mechanotransduction in the inner ear.

## 3 Discussion

TRPC1, TRPC3, TRPC5 and TRPC6 are all expressed in sensory ganglia [10], [31] and TRPC3 and TRPC6 have been shown to be expressed in cochlear hair cells [23]. We previously reported that TRPC3 and TRPC6 DKO mice show selective deficits in sensitivity to innocuous mechanical stimuli and in hearing and vestibular function. TRPC3 and TRPC6 single KO animals, on the other hand, showed unimpaired responses to all sensory stimuli. Therefore it seems that TRPC channels may have a combinatorial role in mediating specific sensory functions. As TRP channels are known to heteromultimerise and are believed to show functional redundancy, the development of TRPC1, TRPC3, TRPC5 and TRPC6 QuadKO animals, generated on a mixed C57BL/6J:129SvEv background at the Comparative Medicine Branch of the NIEHS in North Carolina, by combinatorial breeding of single KO alleles (TRPC1:[6], TRPC3:[15], TRPC5:[22], TRPC6: [7]), has provided us with a novel way of investigating the combined roles of the TRPC channels where monogenic studies may have been unsatisfactory. Using this approach, we have been able to show that knocking out TRPC1 and TRPC5 in addition to TRPC3 and TRPC6 augments specific sensory deficits.

Sensitivity to light touch sensitivity is impaired in QuadKO mice. We found, however, that the impairment was only augmented compared to DKO animals in the cotton swab test while the von Frey withdrawal threshold remained comparable. The cotton swab stimulus is an unequivocally light touch stimulus which is dynamic and thus has different qualities to stimulation with punctate von Frey fibres. Garrison, et al. [11] have previously found that in TRPC1 knockout animals the withdrawal threshold was unaltered but that the responses to subthreshold cotton swab stimuli were impaired. They suggest that this is indicative of a role for TRPC1 involvement in subthreshold mechanical responses which may also be reflected in our multiple KO animals.

Responses to noxious mechanical stimuli were normal in these animals; this is consistent with other data showing TRPC channels do not appear to play a role in mediating noxious mechanosensation [1], [11], [23]. This also highlights a modality specific role for TRPC channels in mediating sensitivity to innocuous, and not noxious, mechanical stimuli. Similarly responses to noxious heat and noxious cold stimuli were unimpaired in QuadKO animals. Although it has been suggested that cold-evoked currents can be produced following heterologous expression of TRPC5, Zimmermann, et al. [34] found behavioural responses in TRPC5 null mice were unaltered. This may be indicative of TRPC5 functioning cooperatively with other TRP channels which are linked to a role in cold sensitivity.

Cochlear hair cells are arranged in a frequency gradient along the basilar membrane in the organ of Corti. They project stereocilia which are deflected by shearing movements between the tectorial and basilar membranes in the organ of Corti in the inner ear, leading to opening of mechanosensitive channels. A similar mechanism of mechanotransduction is found in the vestibular system. Previously, we reported that TRPC3 and TRPC6 were, together, important for normal hearing and vestibular function. These new data support this suggestion and also implicates TRPC1 and TRPC5 in normal hearing function as ABR thresholds were higher in QuadKO animals than DKO animals. In order to determine whether the observed hearing deficits are the result of altered mechanotransduction in the cochlea, mechano-electrical transduction (MET) currents were recorded from cultured OHCs. Since the recordings taken from QuadKO animals were normal, similar both to matching WT controls and previous recordings from OHCs of CD-1 mice [12], [17], we are led to conclude that the loss of TRPC channel function affects the auditory process downstream of the MET channel, though it is possible that function is impaired elsewhere in the cochlea, and that therefore, TRPC channels do not form part of a mechanotransduction complex in the inner ear.

Our earlier electrophysiological work suggests that the role of TRPC channels in mechanosensation is context dependent [23]. TRP channels are notoriously difficult to study in exogenous expression systems because of their function as heteromeric complexes and their interaction with other TRP proteins. Altogether, our data lead us to conclude that the function of TRPC channels involves combined activity of multiple TRPC proteins, something which has been elucidated as a result of the multiple knockout approach. The current work shows that by impairing the function of a further 2 members of the TRPC subfamily we can augment some of the sensory deficits we reported in DKO animals, reinforcing the concept that TRPC channels play a supporting role in in mediating or coordinating mechanosensation. This supports the view that this interaction within the TRPC subfamily is functionally relevant in mechanosensation as interfering with a single TRPC channel leaves behavioural responses unaltered [23] while QuadKO animals show augmented deficits compared to DKO in specific sensory modalities.

The current study substantiates our earlier conclusions that TRPC channels are critical for cutaneous touch sensation. We can now be confident their role in the auditory system is likely to be indirect, as TRPC channels are clearly not primary mechanotransducers. The expression of mechanosensitive currents in neuronal but not non-neuronal cell lines transfected with TRPC3 [23] is intriguing, and suggests that TRPCs may interact with other proteins to form a mechanotransduction complex. TRPC channels are known to interact with a huge list of other proteins and signalling molecules, many of which have already been implicated in mechanosensory roles, including Orai1 which mediates stretch sensitivity in cardiomyocytes and phospholipases which are activated by stretch in a number of sensory systems [3], [9], [25], [26]. This serves to highlight the potentially complex roles these channels may be playing in mechanosensation but also provides an interesting route to identifying other constituents of mechanotransduction complexes.

## 4 Methods

Mice were obtained from the Comparative medicine Branch at the NIEHS, Research Triangle Park, North Carolina, USA. TRPC1, TRPC3, TRPC5 and TRPC6 QuadKO animals were generated on a mixed C57BL/6J:129SvEv background by combinatorial breeding of single KO alleles, TRPC1 [6], TRPC3 [15], TRPC5 [22], TRPC6 [7]. Quad KO mice exhibited generally good health and TRPC3/6 DKO mice were crossed with C57BL/6 mice to generate WT control animals (as previously reported [23]). Both were used for comparison to a TRPC1/3/5/6 QuadKO test group, unless otherwise stated, and mice were aged and sex matched. Behavioural tests, ABRs, MET current recordings from OHCs in organotypic cultures made at postnatal day 2 (P2) and maintained in vitro for 1–2 days and statistical analyses were performed as previously reported [20], [23].

## Conflict of interest

The authors declare no conflict of interest.

## Ethics

All behavioural tests were approved by the United Kingdom Home Office Animals (Scientific Procedures) Act 1986.

## Contributors

JNW designed experiments. JES and KQ performed animal behaviour and analysis. RT and AF performed ABRs and analysis. TD performed MET recordings and TD and CJK performed analysis. JA and LB generated KO mice. All authors contributed to manuscript preparation.
